# Development and Validation of a Radiomics Model Based on 3-Dimensional Endoanal Rectal Ultrasound of Rectal Cancer for Predicting Lymph Node Metastasis

**DOI:** 10.5152/tjg.2023.22257

**Published:** 2023-05-01

**Authors:** Jin Li, Shao-Na Chen, Yun-Yong Lin, Yi-Wen Wu, Wen-Jie Lu, Da-Lin Ye, Fei Chen, Shao-Dong Qiu

**Affiliations:** 1 Department of Ultrasound, the Second Affiliated Hospital of Guangzhou Medical University, Guangzhou, China

**Keywords:** 3-dimensional endoanal rectal ultrasound, radiomic, rectal cancer, lymph node metastasis, nested cross-validation

## Abstract

**Background:**

Development of a radiomics model for predicting lymph node metastasis status in rectal cancer patients based on 3-dimensional endoanal rectal ultrasound images.

**Methods:**

This study retrospectively included 79 patients (41 with lymph node metastasis positive and 38 with lymph node metastasis negative) diagnosed with rectal cancer in our hospital from January 2018 to February 2022. The tumor’s region of interest is first delineated by radiologists, from which radiomics features are extracted. Radiomics features were then selected by independent samples *t*-test, correlation coefficient analysis between features, and least absolute shrinkage and regression with selection operator. Finally, a multilayer neural network model is developed using the selected radiomics features, and nested cross-validation is performed on it. These models were validated by assessing their diagnostic performance and comparing the areas under the curve and recall rate curve in the test set.

**Results:**

The areas under the curve of radiologist was 0.662 and the F1 score was 0.632. Thirty-four radiomics features were significantly associated with lymph node metastasis (*P* < .05), and 10 features were finally selected for developing multilayer neural network models. The areas under the curve of the multilayer neural network models were 0.787, 0.761, 0.853, and the mean areas under the curve was 0.800. The F1 scores of the multilayer neural network models were 0.738, 0.740, and 0.818, and the mean F1 score was 0.771.

**Conclusions:**

Radiomics models based on 3-dimensional endoanal rectal ultrasound can be used to identify lymph node metastasis status in rectal cancer patient with good diagnostic performance.

**Main Points**

- For the first time, the value of a multilayer neural network model based on the radiomics features of 3-dimensional endoanal rectal ultrasound images in the identification of lymph node metastases in rectal cancer patients was discovered.
- Radiomics features based on 3-dimensional endoanal rectal ultrasound images are of great value in the identification of lymph node metastases in rectal cancer.
- Diagnostic performance of multilayer neural network models based on radiomics features is higher than that of radiologists.

## INTRODUCTION

Among various cancers, colorectal cancer is the third most common cancer and the fifth leading cause of cancer-related death in China.^1^ A total of 30%-40% of colorectal cancer is rectal cancer (RC). The status of lymph node metastasis (LNM) in patients with RC has a vital influence on local recurrence, overall survival, and whether the patients need to undergo neoadjuvant radiotherapy (NAT) or chemotherapy.^2^ However, the current medical technology is challenging to accurately predict the LNM status of RC patients before surgery. Meta-analysis studies have shown that even if endoanal rectal ultrasound (ERUS), computed tomography (CT), and magnetic resonance imaging (MRI) are used in combination, the clinical lymph node staging is not certain.^3^

Radiomics is a new concept in recent years. Radiomics is used to solve clinical problems by extracting features from medical images and building machine learning models. Since it was proposed in 2012,^4^ it has played an increasingly important role in cancer research. Using it can improve the accuracy of cancer diagnosis, prognostic assessment, and metastasis prediction.^5^ Machine learning models developed from radiomics features have been widely accepted as reliable tools for predicting clinical events, and has successfully assisted the diagnosis of several malignant tumors, preoperative prediction of LNM status, and prediction of radiotherapy and chemotherapy effects.^6-10^ Multilayer neural network (MLP) is a machine learning model, which belongs to neural network and is a nonlinear statistical classifier.^11,12^ Multilayer neural network has been widely used in the radiomics research. Studies have shown that MLP performs well in the diagnosis of breast tumors, bladder tumors, and gastric tumors.^13-15^ It even showed good results in predicting the incidence of COVID-19.^16^

Three-dimensional endoanal rectal ultrasound (3D-ERUS) is a new technology that can automatically obtain volume data of tissues around the rectum, which can obtain more information than traditional 2-dimensional rectal ultrasound (2D-ERUS). It shows higher accuracy than 2D-ERUS or CT in predicting RC staging and LNM status.^17^

To our knowledge, no study has evaluated whether MLP models based on 3D-ERUS radiomics features can improve LNM state prediction in RC. Therefore, this study aimed to establish a radiomics model for preoperative prediction of LNM status in RC patients.

## MATERIALS AND METHODS

This study involving human participants were reviewed and approved by the Second Affiliated Hospital of Guangzhou Medical University. Written informed consent for participation was not required for this study in accordance with the national legislation and the institutional requirements.

### Study Design

**Figure 1.**
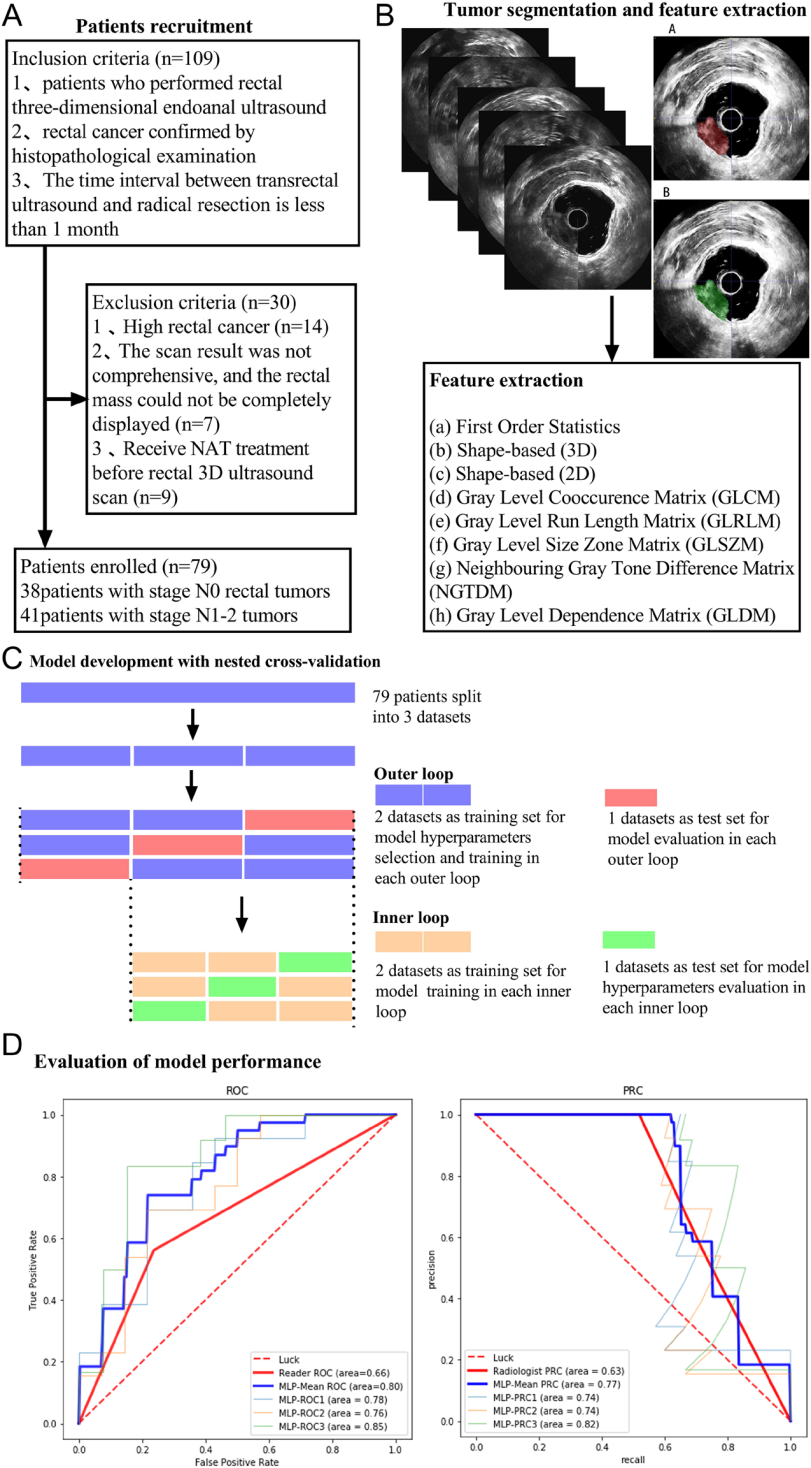
The overview of the study design. (A) Patient recruitment. (B) Tumor segmentation and feature extraction. Manually map the region of interest (ROI) on the largest section of the tumor’s coronal plane. Radiomics features were extracted from ROI masks. (C) Model development with nested cross-validation. Nested cross-validation comprise an inner and outer loop. The inner loop included hyperparameter tuning. The outer loop was performed for the evaluation of model performance. (D) Evaluation of model performance. Receiver operating curve and precision recall curve (P–R curve) analysis were used for model performance evaluation.

The overall design of our study was illustrated in Figure 1, including patient recruitment, tumor segmentation and feature extraction, model development with nested cross-validation, and evaluation of model performance.

### Patients

From January 2018 to February 2022, a total of 109 RC patients who were confirmed through histopathology were retrieved using the Picture Archiving and Communication System work station in our institution. Our inclusion criteria were as follows: (1) 3D-ERUS was performed in the patient prior to surgery; (2) the diagnosis of rectum cancer was confirmed through histopathology analysis; (3) the time interval between 3D-ERUS and radical RC resection was ˂1 month. Exclusion criteria were: (1) the mass belonged to the high RC category and were incapable of undergoing 3D-ERUS examination (n = 14); (2) the mass was too large to be fully included in the ultrasound scan, so the mass could not be completely displayed (n = 7); (3) the NAT treatment was performed before 3D-ERUS (n = 9). A total of 79 patients diagnosed with RC were included in this retrospective study, including 41 (51.9%) males and 38 (48.1%) females. Based on histopathological examination results, these patients were divided into LNM-positive group (stage N1-2) or LNM-negative group (stage N0).

### Three-Dimensional Endoanal Rectal Ultrasound

The ultrasound equipment used was a BK Pro Focus 2202 ultrasound system equipped with the 8820 3D intra-anal probe. The patient received a cleansing enema 2 hours before 3D-ERUS. During the ultrasound examination, 50 mL of warm Coupland was injected into the patient’s rectum through the anus, then the probe was inserted and the tumor was placed in the center of the image, and finally the automatic 3D scanning procedure was started. All 3D-ERUS are acquired with the following parameters: MI 0.86 <1.90, TIS 0.1 <4.0, Res/Hz 2/38 Hz, B Gain 58%, DynRange 71 dB, Harmonic off, persist 1, Edge 3, Noise Reject 15, ACI On, ETC 3.

### Radiologists

**Figure 2.**
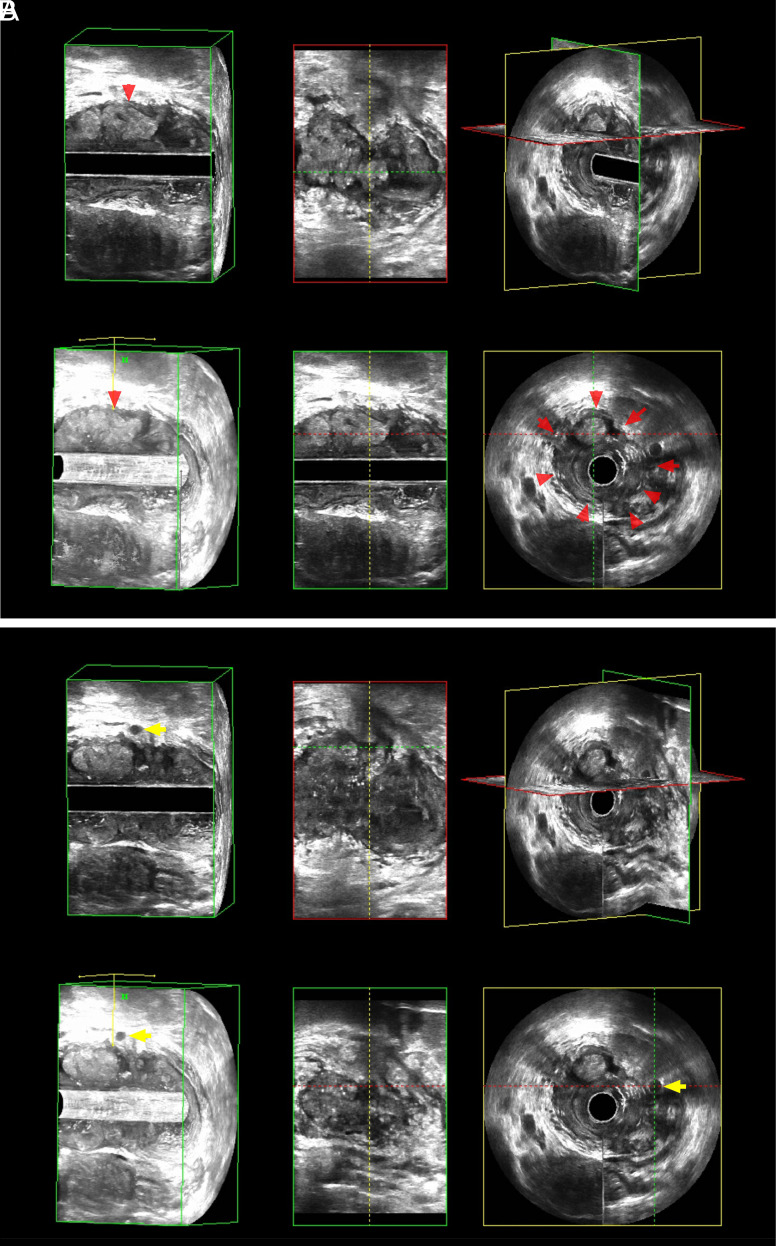
Patient with RC was pathologically confirmed as a representative case of positive LNM. The above-mentioned images are 3D-ERUS scan images, which are 3-dimensional, perspective, sagittal, coronal, cross-sectional, and 3-dimensional sectional views. The red arrow in (A) shows the area of the rectal tumor, and the yellow arrow in (B) shows the lymph nodes around the rectum. 3D-ERUS, 3-dimensional endoanal rectal ultrasound; LNM, lymph node metastasis; RC, rectal cancer.

Three-dimensional endoanal rectal ultrasound images of RC were reviewed by 2 experienced radiologists blinded to pathological information to assess LNM status, as shown in Figure 2. Any disagreement was resolved by consultation.

### Image Segmentation

Region of interests were manually drawn on the largest transverse section of the tumor by 2 experienced radiologists using the ITK-SNAP 3.8 software (https://www.itksnap.org), as shown in Figure 1B. Each disagreement was resolved through discussions. Radiologists were blinded to clinical information.

### Radiomics Feature Extraction

When the 3D-ERUS images segmentation was complete, use the python program to perform radiomics analysis.^18^ A total of 1694 radiomics features in 8 categories were extracted for each patient, including (a) first-order statistics, (b) shape-based (3D), (c) shape-based (2D), (d) gray-level cooccurrence matrix, (e) gray-level run length matrix, (f) gray-level size zone matrix, (g) neighboring gray tone difference matrix, and (h) gray-level dependence matrix.

### Feature Normalization

After feature extraction, the value range of some features is quite different, some ranging from 0 to 10, and some ranging from 0 to 1000. However, we cannot directly assume that features with larger values have greater value to the model. Thus, we normalize values of different features so that all values fall into the same numeric interval.^19^

### Radiomics Feature Selection

Due to the very high dimensionality of radiomics features (n = 1694) compared to the sample size of the study cohort (n = 79), feature selection was necessary to avoid overfitting. To reduce dimensionality, we design a 3-step feature screening procedure. First, use Student’s *t*-test to remove redundant features with small differences. Second, perform correlation analysis on the features. From this step, highly correlated features with correlations above 0.80 are removed. Finally, the least absolute shrinkage and selection operator (LASSO) method is applied for feature screening.

### Model Development

We have developed a preliminary deep learning model, an MLP. Grid-search cross-validation (Grid-search CV) is used for hyperparameter selection during model building.^18^ It is worth noting that in order to avoid the model being too complex, we limit the number of hidden layers of the MLP model to less than 5 layers in Grid-search CV.

### Nested Cross-Validation

Due to the small sample size of this study, it is easy to cause deviations between the research results and the actual situation. Therefore, this study uses 3*3 nested cross-validation to validate the model results.^20,21^ Nested cross-validation contains outer loop and inner loop. In the outer loop, the total sample is split into an outer training set and an outer test set. In the inner loop, the outer training set is split into an inner training set and an inner validation set. The inner loop performs model training on the inner training set, and performs preliminary verification with the inner validation set. The inner loop is performed 3 times, the model hyperparameters are set according to the best validation result. In the outer loop, the model is finally validated using the outer test set and the AUC and F1-score are calculated. The outer loop is performed 3 times to obtain 3 MLP models, and the average AUC value and F1-score are calculated according to the final validation results.

### Statistical Analyses

Feature standardization, selection, and model developing were performed using the Python 3.8.5 (https://www.python.org/). The “scikitlearn” (https://scikit-learn.org/) and “matplotlib” (https://matplotlib.org/) packages were used in this study. Statistical analysis of clinical information was performed using the Statistical Package for Social Sciences version 22.0 software (IBM Corp.; Armonk, NY, USA). Independent samples *t*-test and chi-square test were used to compare the differences in age, gender, histological T/N stage, pathological differentiation, morphology, and histological-type distribution between LNM-positive and -negative groups. Areas under the curve values and F1 scores were used to evaluate the diagnostic performance of deep learning classifiers. *P* < .05 was considered a statistically significant difference.

## RESULTS

### Clinical Characteristics

In this study, 79 patients with RC were finally included, and they were divided into LNM positive group (41 cases) and LNM-negative group (38 cases) according to the LNM status of pathological diagnosis. The clinical data of the 2 groups are shown in Table 1. Among them, the lymph node status reported by the radiologist and pathological T stage were significantly different (*P* < .05).

### Radiologists

Among the 79 patients, radiologist correctly diagnosed 29 lymph node-positive patients and 23 lymph node-negative patients. The AUC is 0.662, the F1-score is 0.632, the specificity is 0.763, the sensitivity is 0.561, and the accuracy is 65.8%.

### Radiomics Feature Extraction and Selection

**Figure 3.**
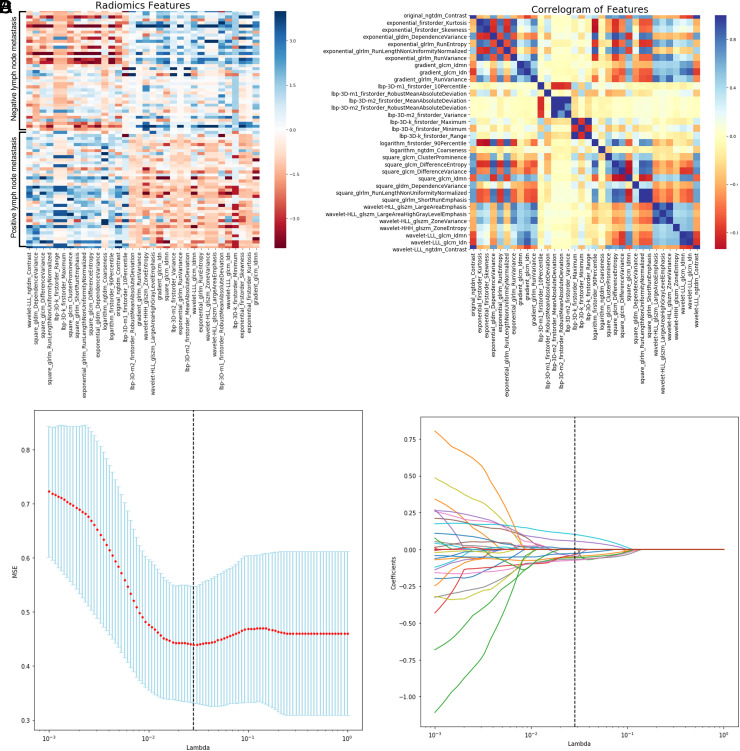
(A) Heat map of the selected features of radiomics classifiers for differentiating between LNM positive and negative. (B) Heat map for correlation analysis of radiomics features. (C) Selection of LNM-associated radiomics features using the LASSO regression. In 10-fold cross-validation, the model has the lowest mean-squared error when the lambda value is 0.02848035868435802. (D) When entering the optimal lambda value, the redundant feature coefficient is returned to 0, and 10 nonzero features are retained. LASSO, least absolute shrinkage and selection operator; LNM, lymph node metastasis.

In the 3D-ERUS images, we extracted a total of 8 categories and 1694 radiomics features. Our results showed that 34 radiomics features were significantly associated with LNM status (*P* < .05) (Figure 3A and B). After the 3-step procedure, 10 features were finally selected for developing the radiomics model (Figure 3B and D).

### Diagnostic Performance of Radiomics Models

**Figure 4.**
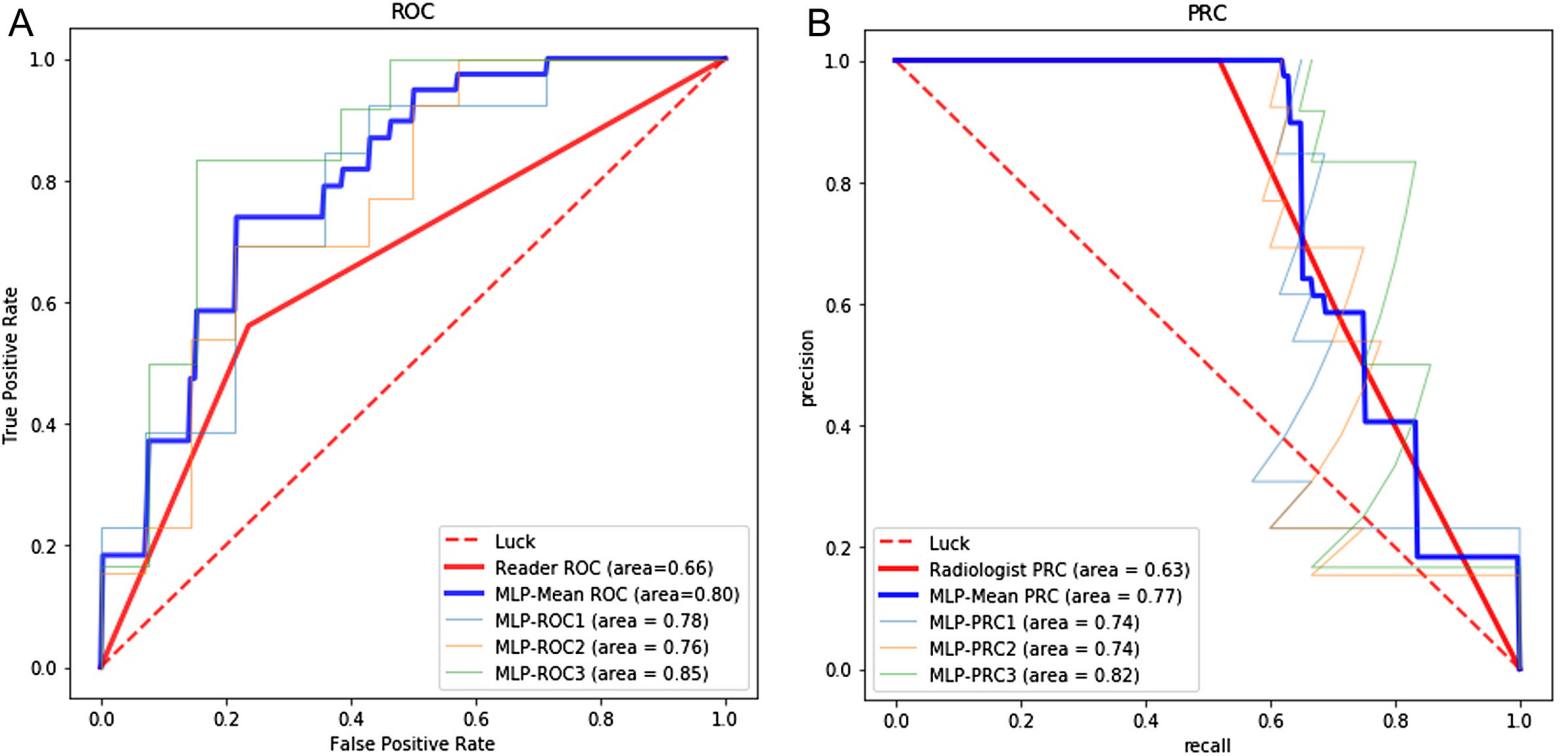
(A) The receiver operating characteristic curve in the prediction of RC LNM. (B) P–R curve in the prediction of RC LNM. LNM, lymph node metastasis; RC, rectal cancer.

The 3*3 nested cross-validation results are shown in Table 2. The AUCs of the 3 MLP models are 0.780, 0.761, and 0.853, and the average AUC is 0.798. The F1 scores of the 3 MLP models are 0.738, 0.740, and 0.818, and the average F1-score is 0.771 (Figure 4A and B). The nested cross-validation results show that the diagnostic performance of the MLP model is higher than that of radiologists.

## Discussion

This study aimed to develop a 3D-ERUS image-based radiomics model to predict LNM status in RC patients. In 3*3 nested cross-validation, the mean AUCs and F1 scores of the MLP models are higher than that of radiologists, showing good effect in predicting LNM status in RC patients.

The LNM status in RC patients has a vital influence on local recurrence, overall survival, and whether patients need to undergo NAT. The LNM status of RC patients is a reference indicator for deciding whether to perform NAT. For patients with advanced RC, surgical resection after NAT treatment could reduce the risk of local recurrence by 50%-61% compared to surgery alone.^22^ Therefore, accurate preoperative assessment of LNM status is crucial for optimizing treatment regimens and prognostic prediction. However, it remains a challenge to assess LNM status before surgery.

In this study, radiologists had poor results in predicting LNM status in RC patients using 3D-ERUS images (AUC = 0.662), which was consistent with previous studies.^3^ This may bring wrong information to clinicians, leading to the wrong choice of treatment options.

Radiomics can extract information from images to detect differences that cannot be detected by visual inspection.^4,5^ Previous studies have predicted the LNM status of RC patients based on the imaging characteristics of 2D-ERUS, CT, and MRI.^8,23-27^ The AUC of the nomogram model based on radiomics features for predicting LNM status in RC patients was 0.77, 95% CI 0.67-0.86.^28^ A nomogram model was constructed by combining ERUS, CT, and shear wave elastography images for predicting LNM status in RC patients with a concordance index of 0.857.^24^ However, previous research results have only performed single-pass validation or single-layer cross-validation, and the results are highly dependent on the division of test sets. Nested cross-validation can avoid this problem, especially when making analytical decisions or adjusting model parameters after observing analytical results, which may produce overestimated results.^29^ Nested cross-validation is widely used in machine learning and deep learning.^20,30-32^ Compared with a simple cross-validation, nested cross-validation can reduce overfitting and limit optimism bias. Especially in relatively small samples, nested cross-validation procedure provides an almost unbiased estimate of the true error.^29,33^ Therefore, this study developed an MLP model to predict LNM status in RC patients and performed 3*3 nested cross-validation to validate the model’s diagnostic performance.

In this study, 1694 radiomics features were extracted from 3D-ERUS images of patients with RC. To avoid model overfitting, 1684 (99.4%) were eliminated using independent sample *t*-test, feature correlation coefficient analysis, and LASSO regression analysis, and only 10 best features were finally preserved. For a large number of radiomics features extracted from a relatively small sample, LASSO regression analysis can avoid model overfitting.^34^ Based on the above 10 best features, we built the MLP model. The MLP model is a preliminary deep learning model and nonlinear classifier. It contains multiple hidden layers, and each hidden layer contains multiple neurons. With the cooperation of multiple neurons, it can show the complex relationship between dependent variables and independent variables.^11,12^ The MLP models have been effectively applied to the diagnosis of liver cancer and breast cancer, to predict LNM status in breast cancer patients, and to assess the risk of cardiovascular disease in patients.^35-37^ It is worth noting that the increase of hidden layers will also increase the complexity of the model. Studies have shown that an overly complex model can easily lead to overfitting and reduce the performance of the model for unfamiliar objects. To avoid this situation, this study used grid-search CV to optimize the model hyperparameters while limiting the number of hidden layers in the model.

In order to verify the actual effect of the model applied to the clinic, the model was validated. Due to the small sample size of this study, it is easy to cause model overfitting. In layman’s terms, overfitting means that an artificial intelligence (AI) model learns in a way that is only applicable to training samples, and no longer generalizes to the entire population.^29,38^ This means that the model has extremely high performance on the training set and extremely low performance on the validation and test sets. Therefore, this study uses 3*3 nested cross-validation to verify the model results. The 3*3 nested cross-validation results of the MLP models show that the 3 models have better performance on the test set and validation set, respectively. The diagnostic performance of the test set and validation set is lower than that of the training set, but the gap is within an acceptable range. It is worth noting that for each model, the validation and test sets are external datasets, which indicates that the model has a low degree of overfitting. Based on the mean AUC and mean F1-score, the MLP models showed good diagnostic performance in multiple validations, and all were larger than the radiologist’s AUC, showing good reliability and repeatability.

Nevertheless, our study has several limitations: First, our relatively small sample size may cause unstable results, even if we use nested cross-validation. Second, this study lacks multi-institution verification of radiomics characteristics. Finally, instead of 3D analysis of the entire lesion volume, we performed a 2D analysis of the region of interest in the largest slice of the lesion cross-section. This method is less labor intensive but less sensitive to intertumoral changes.

## CONCLUSION

In conclusion, the radiomics features based on 3D-ERUS images are of great value for identifying the LNM status of RC patients, and the diagnostic performance of the MLP models constructed based on it are better than that of radiologists. Multicenter retrospective validation and prospective randomized clinical trials should be performed in subsequent studies to obtain high-level evidence for the clinical application of this radiomics model.

## Figures and Tables

**Table 1.** Comparison of Clinical Data of Patients with LNM-Positive and -Negative Groups

	Negative Group (n = 38)	Positive Group (n = 41)	*P*
Age	63.63 ± 9.55	62.20 ± 14.96	.610
Gender			
Male	20 (52.6%)	21 (51.2%)	.900
Female	18 (47.4%)	20 (48.8%)	
BMI	22.50 ± 3.25	22.33 ± 2.37	.787
CEA	3.24 (3.47)	5.84 (9.93)	.077
Ca 19-9	8.37 (11.95)	9.00 (12.29)	.898
Tumor size (cm^3^)	3.52 (3.15)	3.28 (2.56)	.517
Echo			
Hypo echo	10 (26.3%)	18 (43.9%)	.122
Medium echo	16 (42.1%)	17 (41.5%)	
Hyper echo	12 (31.6%)	6 (14.6%)	
Border			
Smooth	0 (0.0%)	0 (0.0%)	
Hazy	38 (100.0%)	41 (100.0%)	
Blood flow			
Stage 0	0 (0.0%)	0 (0.0%)	.063
Stage 1	14 (36.8%)	11 (26.8%)	
Stage 2	10 (26.4%)	20 (48.8%)	
Stage 3	14 (36.8%)	10 (24.4%)	
T stage of tumor reported by ultrasound			
uT1	2 (5.3%)	0 (0.0%)	.641
uT2	2 (5.3%)	3 (7.3%)	
uT3	28 (73.7%)	30 (73.2%)	
uT4	6 (15.8%)	8 (19.5%)	
Lymph node status reported by ultrasound			
uN0	29 (76.3%)	18 (43.9%)	.006
uN1	9 (23.7%)	23 (56.1%)	
Boundary with surrounding organs			
Clear	32 (84.2%)	34 (82.9%)	1.000
Not clear	6 (15.8%)	7 (17.1%)	
Artery V_max_ (cm/s)	13.9 (8.11)	14.57 (13.32)	.887
Artery RI	0.71 (0.95)	0.69 (0.17)	.933
Pathological T staging			
T1	2 (5.3%)	0 (0.0%)	<.001
T2	19 (50.0%)	4 (9.8%)	
T3	12 (31.6%)	21 (51.2%)	
T4	5 (13.2%)	16 (39.0%)	
Tumor differentiation			
Well differentiated	0 (0.0%)	6 (14.6%)	.087
Moderate to well differentiated	29 (76.3%)	26 (63.4%)	
Moderate differentiation	4 (10.5%)	3 (7.3%)	
Poorly differentiated	5 (13.2%)	6 (14.6%)	
Tumor appearance			
Polyp type	22 (57.9%)	17 (41.5%)	.263
Ulcer type	12 (31.6%)	20 (48.8%)	
Flat type	3 (7.9%)	4 (9.8%)	
Sessile type	1 (2.6%)	0 (0.0%)	
Histological type			
Adenocarcinoma	34 (89.5%)	33 (80.5%)	.457
Mucinous adenocarcinoma	4 (10.5%)	6 (14.6%)	
Signet ring cell carcinoma	0 (0.0%)	2 (4.9%)	

BMI, body mass index; CEA,carcinoembryonic antigen; Ca199, Carbohydrate antigen199; LNM, lymph node metastasis.

**Table 2.** Area Under the Curve Value of Radiomics Model in Nested Cross-Validation

	Loop 1	Loop 2	Loop 3	Mean
Training set	0.835	1.000	0.975	0.937
Validation set	0.773	0.832	0.797	0.801
Test set	0.780	0.761	0.853	0.798
